# Synthesis and Characterization of PEGylated Liposomes and Nanostructured Lipid Carriers with Entrapped Bioactive Triterpenoids: Comparative Fingerprints and Quantification by UHPLC-QTOF-ESI^+^-MS, ATR-FTIR Spectroscopy, and HPLC-DAD

**DOI:** 10.3390/ph18010033

**Published:** 2024-12-31

**Authors:** Carmen Socaciu, Florinela Fetea, Mihai Adrian Socaciu

**Affiliations:** 1 Faculty of Food Science and Technology, University of Agricultural Sciences and Veterinary Medicine, 400372 Cluj-Napoca, Romania; florinelafetea@usamvcluj.ro; 2 Department of Biotechnology, BIODIATECH—Proplanta Research Centre for Applied Biotechnology in Diagnosis and Molecular Therapy, 400478 Cluj-Napoca, Romania; 3 Faculty of Medicine, University of Medicine and Pharmacy “Iuliu Hatieganu”, 400347 Cluj-Napoca, Romania

**Keywords:** pentacyclic triterpenoids, PEGylated liposomes, nano lipid carriers, diffraction light scattering, ATR–Fourier-transform infrared spectroscopy, high-performance liquid chromatography, mass spectrometry

## Abstract

**Background/Objectives**: Pentacyclic triterpenoids, as bioactive phytochemicals, have proven to exhibit significant bioactivity (antioxidant, anti-inflammatory, hypoglycemic, and anticancer) and low cytotoxicity. This study developed convenient methods for extracting and characterizing a birch bark extract enriched in pentacyclic triterpenoids (betulin, betulinic acid, and lupeol) and entrapped in two bioavailable nanoformulations. The performance of ATR-FTIR spectroscopy as a cost-effective and non-destructive method was evaluated comparatively with accurate HPLC-based methods. **Methods**: The bark extract and pure betulin or betulinic acid were used to obtain PEGylated liposomes and nano lipid carriers (NLCs). Their size was characterized by light scattering diffraction. UV–Vis spectrometry was applied as a preliminary evaluation (1), as well as UHPLC-QTOF-ESI^+^-MS for structure identification (2), ATR-FTIR spectroscopy (for semi-quantitative evaluation) (3), and HPLC-DAD for an accurate quantification of each component, either in the organic solvents or in the nanoformulations (4). **Results**: The PEGylated liposomes had smaller sizes, and higher entrapment efficiency, significantly correlated between the three analytical methods. The performance of ATR-FTIR spectroscopy was positively correlated with HPLC-DAD data and confirmed the potential of this cheaper and reliable semi-quantitative method to evaluate the entrapment efficiency of TTs in liposome and NLC nanoformulations. **Conclusions**: The results recommend using liposomal nanoformulations for the entrapment of bioactive terpenoids and their characterization by ATR-FTIR after validation by HPLC-DAD. The ATR-FTIR spectroscopy also offers the possibility of screening in a short time different recipes of nanoformulations as well as their stability and bioavailability, which is useful for investigations in vitro and in vivo, which may confirm their efficacy as therapeutic agents.

## 1. Introduction

**Figure 1:**
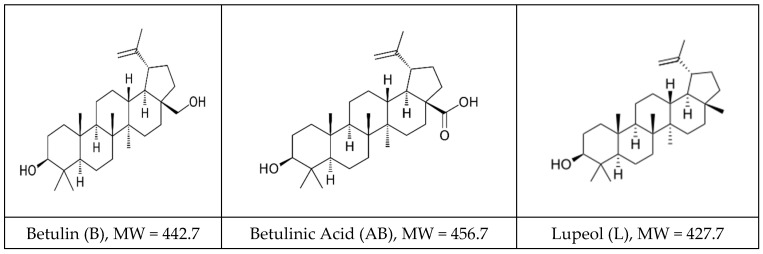
Most representative pentacyclic lupane-type triterpenoids: structure, molecular weight [MW], and maximal UV absorption.

Pentacyclic triterpenoids are an important family of bioactive phytochemicals and are classified in different classes, lupane-type (lupeol, betulin, and betulinic acid), oleanane-type (oleanolic acid, ß-amyrin, and erythrodiol), and ursane-type (ursolic acid, α-amyrin, and uvaol). The lupane family is the most known, with betulin, betulinic acid, and lupeol showing diverse therapeutic effects. The outer bark of *Betula Pendula Roth* (birch tree) contains betulin (B) as a major component along with betulinic acid (AB) and lupeol (L), as shown in Figure 1.

These pentacyclic triterpenes (TTs) have proven to have various biological functions, including antiviral, anticancer, antioxidant, anti-inflammatory, and hypoglycemic activities, demonstrating their actions as pharmacological agents and potent anticancer molecules [1,2,3,4]. The interest in lupane-type molecules has increased since betulinic acid and betulin proved to induce cytotoxicity against human melanoma lines in vitro and in vivo [5,6,7]. A recent review systematically summarized the chemical structures, plant sources, bioactivities, absorption, metabolism, and oral bioavailability of TTs, as well as their self-assembly properties, suggesting that their derived nanostructures are not only efficient in oral forms [8]. Meanwhile, chemical modifications and new functional delivery systems were realized to attenuate their hydrophobicity and poor absorption, and to improve their bioavailability and bioactivity [9]. Some of these molecules are recognized by the US Food and Drug Administration as Generally Recognized as Safe (GRAS), considered as bioactive compounds and excipients for other phytochemicals or drugs, especially for topical routes of administration [10].

Betulinic acid (AB), found mainly in white or silver birch, showed potent pharmacological properties, low side effects, and a prominent efficiency in cancer therapy. The anti-proliferative effect of AB is explained by the inhibition of topoisomerase I and II in tumor cells (1), changes in the mitochondrial membrane potential and mitochondrial apoptosis (2) and anti-angiogenesis (3), and DNA polymerase inhibition, the activation of caspases, and the production of reactive oxygen species, as recently reviewed [6].

In the last decades, nanoparticle-based drug delivery has been extensively exploited for cancer treatment by the oral, parenteral, and topical delivery of drugs due to their improved stability and biocompatibility, enhanced permeability and retention, and precise targeting. Multiple forms of lipid nanoparticles exist, including liposomes, solid lipid nanoparticles, nanostructured lipid carriers (NLCs), micro- and nanoemulsions, phytosomes, lipid-coated nanoparticles, and nanoassemblies. Recently, updated information about nanoformulations for the delivery of TTs in anticancer therapies has been reported [1,7]. Meanwhile, it has been shown that some triterpenoids can be self-assembled as 3D structures or bilayer structures and can incorporate different fluorophores as well anti-cancer agents at physiological and lower pH, making them useful as drug delivery vehicles [11].

To enhance AB’s solubility, half-life, and efficacy by using nanoscale drug delivery systems, several systems, including polymeric nanoparticles, magnetic nanoparticles, liposomes, polymeric conjugates, nanoemulsions, cyclodextrin complexes, complexes with carbohydrate nanoparticles, and carbon nanotubes, have been developed [12,13]. Different liposomal systems incorporating AB were obtained (with sizes of around 100–150 nm), which induce cell cytotoxicity and apoptosis, the destruction of the mitochondrial membrane potential, and the in vivo inhibition of tumors in mice by intratumoral injection [14,15]. The PEGylated AB liposomes, with sizes of around 142 nm, can effectively accumulate in tumor tissues, had an increased drug release and tumor inhibitory effect, compared with those of free AB, and represent a better alternative for cancer therapy [16].

Due to their unique size-dependent properties, nanostructured lipid carriers (NLCs), a second generation of solid lipid nanoparticles (SLNs), contain a mixture of physiologically similar lipids and are well-tolerated [16,17,18]. Their ability to incorporate lipophilic drugs offers a possibility to create new prototypes with an increased bioavailability for drug delivery, along with controlled and site-specific drug delivery. NLCs produced by a melt-emulsification and ultrasonication method have numerous applications in both the pharmaceutical and cosmetic industries due to their ease of preparation, their feasibility of scale-up, biocompatibility, non-toxicity, and enhanced targeting efficiency, and the possibility of site-specific delivery via various routes of administration [19,20].

To evaluate the composition and stability of terpenoids like AB, B, or L, in organic solvents or in different nanoformulations, several analytical methods were applied, from gas or liquid chromatography coupled with UV–Vis detection or mass spectrometry (HPLC-DAD or UHPLC-MS), infrared or Fourier-transform infrared spectroscopy (FTIR), to near-infrared (NIRS) or Raman spectroscopy. IR spectroscopy has been used successfully for qualitative or semi-quantitative analysis in various fields including the pharmaceutical and food industries, and in biological evaluation. A simple, rapid, and non-destructive method for the determination of betulin in the outer birch bark was developed using NIRS, combined with HPLC-DAD, to determine the quality and to quantify the betulin content of the outer birch bark [21]. Vibrational FTIR and FT-Raman spectroscopy was applied for a qualitative and semi-quantitative analysis of *Betula pendula* bark samples dissolved in methanol in parallel with GC-MS analysis, revealing two major compounds, betulin (59%) and lupeol (41%), and traces of betulinic acid, with specific absorbances of the –COOH group at 1681 and 1718 cm^−1^ [15]. Moreover, complexes of betulin–cyclodextrins have been prepared and analyzed using FT-Raman spectroscopy [22].

Recently, NLC with entrapped betulin were synthesized using the emulsification/solidification method, exhibiting a spherical shape and particle sizes of around 183 nm, a polydispersibility index (PDI) of 0.142, and a high zeta potential of −38.64 mV, reflecting a relative stability of the nano system. The drug loading into NLC had entrapment efficiencies (EEs) from 47% to 87%, useful for a topical application in psoriasis treatment [23].

The Attenuated Total Reflection ATR-FTIR provides a rapid, cost-effective, and attractive platform without the need for any sample preparations, as solutions or after evaporation on the ATR diamond crystal without further preparation [24]. Considerable research data have demonstrated the superior performance of FTIR in comparison to conventional techniques, including accessible disease diagnostics, although its routine application within clinical laboratories remains elusive [25]. Recent developments and updates for the qualitative and quantitative analysis of herbal medicine using FTIR were reviewed, useful in herbal drug development, in production for process monitoring, or in quality control laboratories [26,27].

HPLC-MS is a highly sensitive method for the quantitative determination of triterpenoids in plant extracts based on a combination of HPLC with atmospheric pressure chemical ionization and tandem mass spectrometric detection, with detection limits of 0.7–1.8 μg/L [28]. Recently, the quantitative analysis of pentacyclic triterpenoids and the separation of ten major molecules in different plants, including birch bark, were reported, using supercritical fluid chromatography–tandem mass spectrometry (SFC-MS/MS) on silica-based reversed stationary phases [29].

This study developed convenient methods for the extraction and characterization of a birch bark extract enriched in pentacyclic triterpenoids (betulin, betulinic acid, and lupeol) and entrapped in two bioavailable nanoformulations (PEGylated liposomes and NLC). The size characterization of two types of nanoformulations which can entrap bioactive pentacyclic triterpenoids (pure B or AB and a birch bark extract) was achieved by diffraction light scattering, and their entrapment efficiency used the UV–Vis spectrometry successively as a preliminary evaluation (1), UHPLC-QTOF-ESI^+^-MS for structure identification (2), ATR-FTIR spectroscopy for fingerprinting and semi-quantitative evaluation (3), and quantification by HPLC-DAD of each component from the bark extract comparative to pure standards of AB and B (4). Finally, the performance of ATR-FTIR spectroscopy as a cost-effective and non-destructive method is evaluated comparatively with accurate HPLC-based methods.

## 2. Results

### 2.1. Preparation and Characterization of the TT Extract by UV–Vis Spectrometry

The TT extract was obtained in three steps by successive extractions with different solvents, as described in the Section 4. A preliminary evaluation of the TT extract was carried out by UV spectrometry (200–320 nm) in the solvent mix EtOH:DMSO (3:1) comparative to calibrations with pure standards of AB and B measured at four different concentrations, from 2 to 20 mg/mL. The total concentration of terpenoids in the TT extract was evaluated from its maxima absorption at 212 nm in the UV spectra. The mean value from a triplicate measurement indicated a concentration of 28.35 mg/mL. This was a rough evaluation, before its quantification by HPLC-DAD.

### 2.2. Identification and Quantification of TT Components by UHPLC-QTOF-ESI^+^-MS and HPLC-DAD

**Figure 2:**
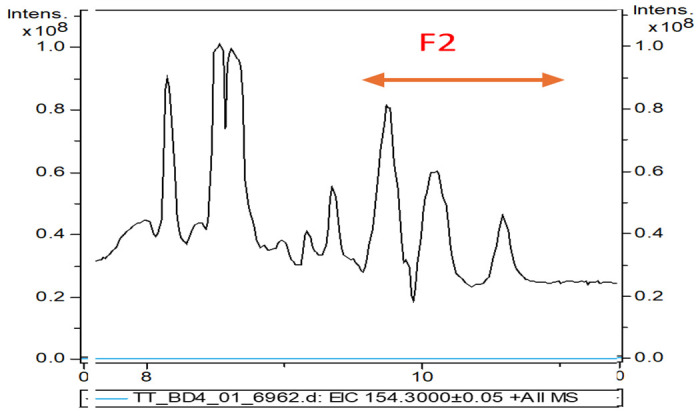
The LC-MS chromatogram of the initial raw TTs extract including the fraction F2 collected between 9.5–11 min and used as a standardized TTs in further experiments. Fraction F2 includes B (major peak), followed by AB and L (minor peak).

The same TT extract was characterized by a UHPLC-QTOF-ESI^+^-MS analysis. Figure 2 shows the LC chromatograms of the initial raw bark extracted in acetonitril (Figure 2) and fraction F2 collected between 9.5–11 min, representing the TT extract to be used in further experiments (Figure 2).

**Figure 3:**
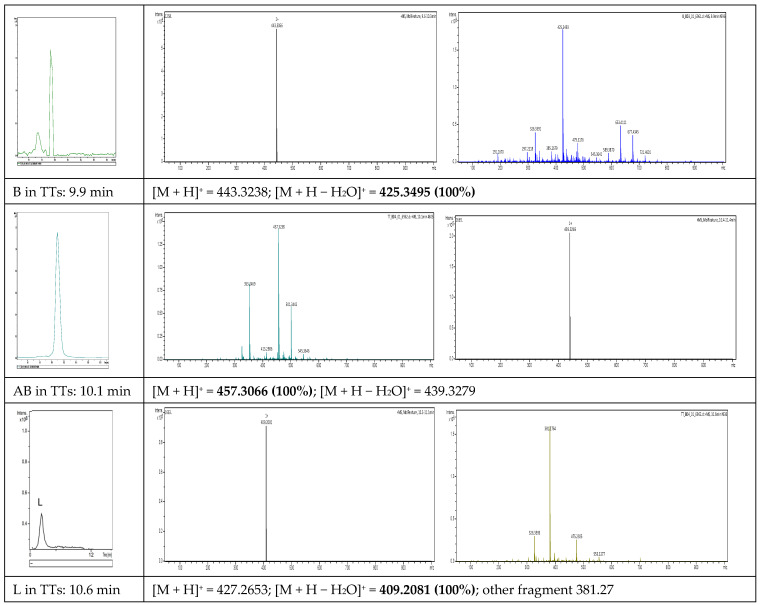
Identification of the three components of the TT extract (B, AB, and L) using EIC and the specific MS fragments.

In fraction F2, we identified three major terpenoids, namely, betulin (**B**), betulinic acid (**AB**), and lupeol (**L**). Their identification was made by mass spectrometry, based on specific precursor ions and fragments, as shown in Figure 3. By an extract ion chromatogram (EIC), each component was characterized, as shown on the left side of Figure 3. The mass spectra of precursors [M + H]^+^ and other fragments, mainly [M + H − H_2_O]^+^, are mentioned.

Betulin (B) (min 9.9) was identified by its main precursor with *m*/*z* = 443.3238 Da and the dominant fragment with *m*/*z* = 425.3495 Da (100%), while betulinic acid (AB) eluted at 10.1 min, the precursor ion had *m*/*z* = 457.3066 Da (100%), and the fragment [M + H − H_2_O]^+^ with *m*/*z* = 439.3279 Da. Luteol was identified at min 10.6 and had three specific fragments at 427.2653 Da (low abundance), 409.2081 Da (100%), and 381.27 Da. In parallel, calibrations were made with four different concentrations of betulin (1 to 10 mg/mL) by HPLC-DAD; see details in Section 2.5 and Supplementary File S1 (HPLC-DAD chromatograms and calibration curve with pure betulin (1–10 mg/mL)). Considering that the protocol of HPLC-DAD, the solvents, and the gradient protocol were different (see Section 4), the maximal absorption was at 212 nm and the retention times in the TT extract were different in this case, namely, B-Rt = 5.5 min, AB-Rt = 5.9 min, and L-Rt = 6.35 min. The ratio of B:AB:L was determined to be 55:35:10 according to peak areas ratios. The concentration of the stock extract of TTs expressed in B equivalents was estimated to be 25.4 mg/mL. This standardized extract TTs were used to build all nanoformulations and compared with similar formulations containing pure AB and B (as positive controls).

### 2.3. Characterization of the Nanoformulations with Entrapped AB and TTs

**Figure 4:**
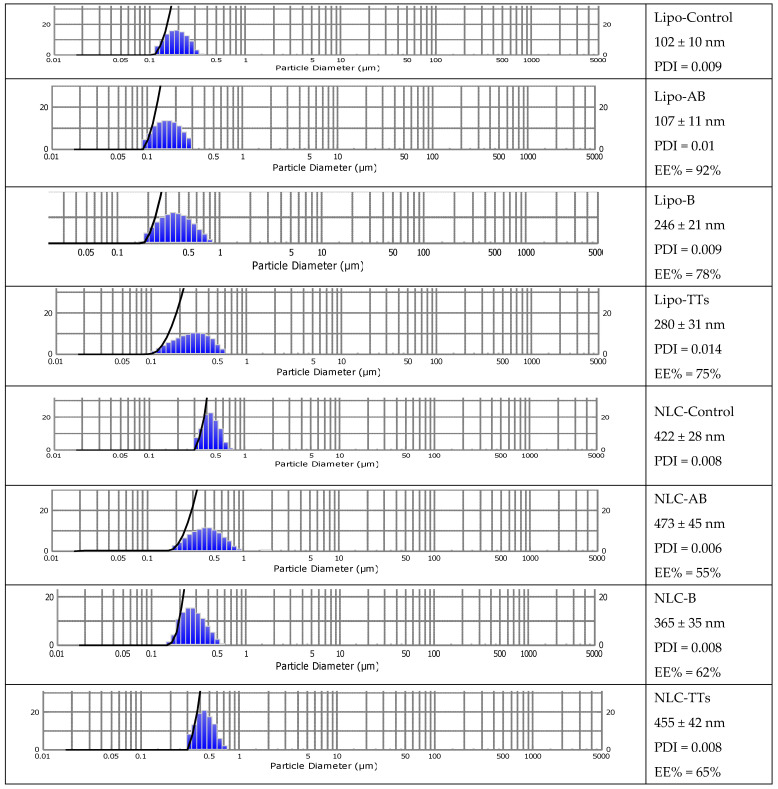
Mean sizes of Lipo- and NLC-nanoformulations containing entrapped AB, B, and TTs comparative to controls, as determined by DLS. Size distribution diameters (mean ± SD) (nm) of the formulations, and the PDI value and the encapsulation efficiency (EE%) are included.

Figure 4 includes the size distribution and diameters (mean ± SD (nm) and dispersibility index (PDI) of all Lipo- and NLC-formulations. The entrapment efficiency (EE%) is also mentioned. It was determined by UV spectrometry after the elimination (by ultrafiltration) of the non-entrapped terpenoids; for details, see Section 4.

As shown, the population of nanoparticles had sizes between 102–473 nm, depending on the type of nanostructure and the terpenoid type. The polydispersibility index (PDI) showed a good distribution of nanoparticles in all cases. The smaller Lipo- structures entrapped AB with higher a EE% (92%), while B and TTs had larger sizes (above 200 nm) and EE% values of 78 and 75%, respectively. All NLC-formulations had larger sizes and showed the formation of cloudy networks of lipid nanostructures, with an increased tendency for aggregation compared to Lipo- structures. NLC-AB had a mean diameter of 473 nm and EE% = 55%, while entrapped B and TTs showed sizes of 365 ± 35 nm and 455 ± 42 nm, respectively, while the EE% was 62% and 65%, respectively. The stability of both types of nanoformulations was high, with constant sizes, as checked for one month of storage at 4 °C. Significant correlations were found between microscopic evaluation and the DLS measurements, as checked previously [30].

### 2.4. ATR-FTIR Fingerprinting and Semi-Quantitative Evaluations of AB, B, and TT Before and After Entrapment in Lipo- and NLC Nanoformulations

**Figure 5:**
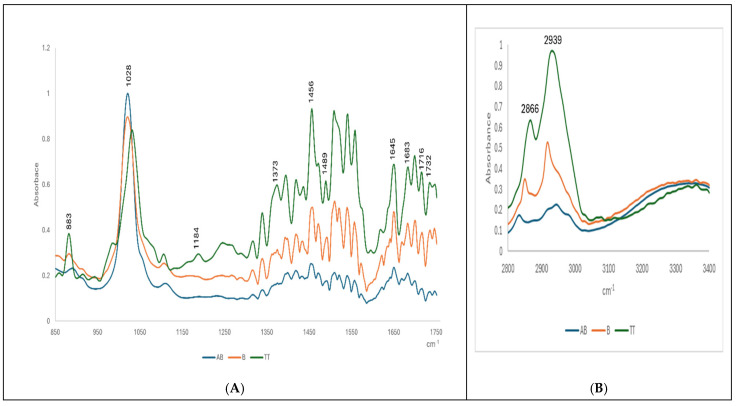
The ATR-FTIR superposed fingerprints of AB and B compared to TT extract, after evaporation on the diamond cell: (**A**) the spectra were recorded in region 850–1750 cm^−1^ and in the region 2800–3400 cm^−1^ (**B**).

The ATR-FTIR fingerprinting of the TT extract compared to AB and B was carried out either in the solvent mix and in the evaporated sample on the diamond cell (see Supplementary File S2A). Figure 5A,B show the ATR-FTIR superposed fingerprints of AB and B compared to the TT extract, after evaporation. The spectra were recorded in the region 850–1750 cm^−1^ (Figure 5A) and 2800–3400 cm^−1^ (Figure 5B).

The spectral pattern was rather similar for all three molecules, exhibiting the almost same main band positions and relative intensities at common wavenumbers corresponding to specific vibrations of polar and non-polar bounding. Table 1 includes details about these vibrational bands, corresponding to different molecular vibrations.

The assignments were similar with other reported data [15,32], as can be seen in columns 4–7. The peaks of the region were at 2800–3400 cm^−1^. We identified shifts from 2852 cm^−1^ and 2945 cm^−1^ (specific to AB), to 2823 and 2922 cm^−1^ (for B), and 2866.2 and 2939.5 cm^−1^ for TTs. These strong bands correspond to the symmetric and anti-symmetric stretching in the CH_2_ groups of alkyl chains, while the band centered at 1741 cm^−1^ corresponds to the stretching vibrations of the ester carbonyl groups. The band centered at 1650 cm^−1^ is assigned to C=O stretching, and the scissoring vibrations of the CH_2_ groups are represented by the band at 1465 cm^−1^.

The ATR-FTIR absorbance of the TT extract compared to pure betulin and betulinic acid revealed specific molecular fingerprints of triterpenes, based on the main bands in both regions (850–1750 cm^−1^ and 2800–3400 cm^−1^). We identified 11 specific bands, as presented in Table 1; the spectral feature is observed for the band at 1716–1735 cm^−1^ attributable to the ν(C=O) mode, that is prominent in the bark spectrum [15]. In agreement with the literature data, stretching vibrations of C=O were identified from 1028 to 1180 cm^−1^ Characteristic alkyl (R-CH2) stretching modes from 2850 to 3000 cm^−1^ were observed, in accordance with other references [31].

For a semi-quantitative evaluation of these molecules, either as free molecules in a solvent mix or in nanoformulations, calibration curves for B were built based on their ATR-FTIR spectra recorded in solution at four different concentrations (2, 4, 10, and 20 mg/mL). For each of the 11 wavenumbers (mentioned in Table 2), the calibration curve was built, and the equation together with the R^2^ (coefficient of determination) was mentioned (see Supplementary File S3).

**Figure 6:**
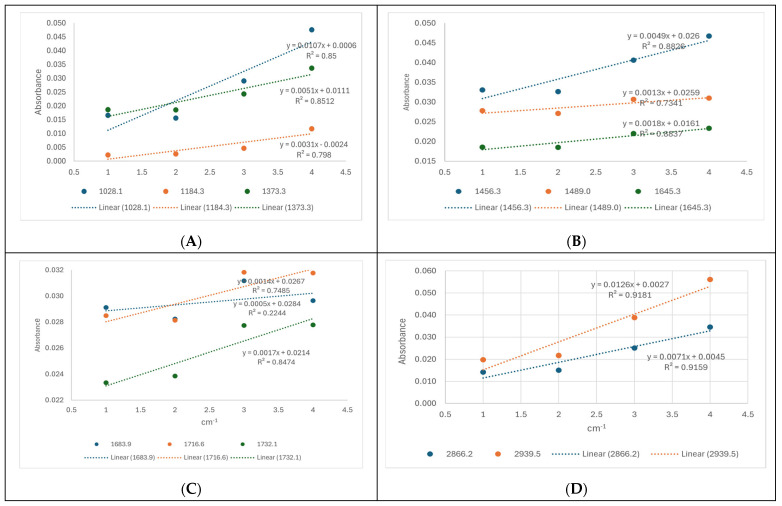
Dose-absorbance curves recorded for four dilutions of TTs in EtOH:DMSO (3:1) at 11 wavenumbers, as mentioned in the legend. Dilution 4 = stock TTs diluted 2.5x with EtOH: DMSO (3:1). Dilutions 3, 2, and 1 = successive dilutions of 4 (1.5, 2, and 4 times). (**A**) 1028.1; 1184.3; 1373.3 cm^−1^. (**B**) 1456.3; 1489.0; 1645.3 cm^−1^. (**C**) 1683.9; 1716.6; 1732.1 cm^−1^. (**D**) 2866.2; 2939.5 cm^−1^.

The highest values of R^2^ and the correlation between concentration/absorbance were identified at 2866.2 and 2939.5 cm^−1^ for B standards. The same correlation was obtained from the TT extract using four dilutions (dilution nr 4 representing the stock solution diluted 2.5 times in EtOH:DMSO), as shown in Figure 6. All dilutions were made in the same solvent mix and the highest R^2^ values (above 0.91) were found at 2939.5 and 2866.2 cm^−1^ (Figure 6D).

According to the equation for B (y = 0.0034x + 0.0199; R^2^ = 0.992) at 2939.5 cm^−1^, the concentration of TT dilution 4 was determined to be 10.32 mg/mL. Therefore, the stock solution of TTs was calculated to be 2.5 times this value, namely, 25.8 mg/mL. This value was compared with the evaluation made by HPLC-DAD (25.4 mg/mL) and UV spectrometry (28.35 mg/mL), as presented in Section 2.1 and Section 2.2. In conclusion, the ATR-FTIR evaluation of the TT extract gave similar results with the other methods and can be considered reliable for quantitation in further experiments, considering the specific wavenumbers 2866.2 and 2939.5 cm^−1^.

**Figure 7:**
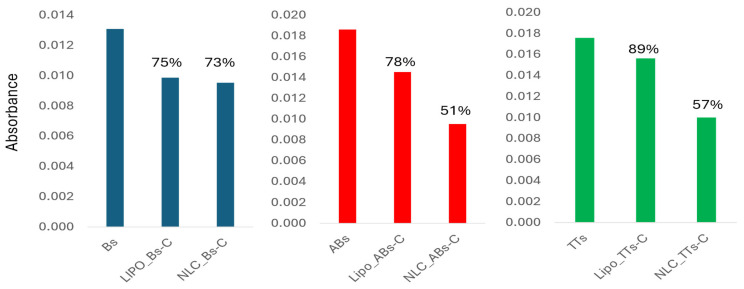
The EE% values calculated from the ATR-FTIR spectra at 2939.5 cm^−1^, considering the absorbance of the entrapped ABs, Bs, and TTs in Lipo- and NLC-formulations with subtracted values from controls (C).

These data were useful to make the evaluation of the EE% values of AB, B, and TTs in the Lipo- and NLC-formulations, considering specifically the absorbance values recorded at 2866.2 and 2939.5 cm^−1^. In the Supplementary File S2B, the ATR-FTIR spectra of Lipo-entrapped TTs, B, and AB compared to the Lipo-control are presented, recorded after dissolving in the solvent mix (s) or after evaporation. In the Supplementary File S2C, similar ATR-FTIR spectra of NLC-entrapped TTs, B, and B to the NLC-control are presented, recorded after dissolving in the solvent mix(s) or after evaporation. In both cases, considering their absorbances at wavenumbers 2866.2 and 2939.5 cm^−1^, the entrapment efficiency was calculated. Figure 7 shows comparatively the EE% values calculated for the entrapped AB, B, and TT in Lipo- and NLC-formulations and the organic solutions used for entrapment.

The EE% values shown in the first columns (blue) were similar in the Lipo- and NLC-formulations, and were around 74% compared to the initial concentration of Bs. The EE% values shown in the red columns were 78% in Lipo-ABs-C compared to 51% for NLC-ABs-C. For TTs, the EE% values were 89% vs. 57% in the Lipo- versus NLC-formulations, and for B in solution (Bs) compared to B entrapped in Lipo- and NLC-formulations. As a conclusion, Lipo-formulations showed similar EE% values for all terpenoids while NLC-formulations showed inferior values, especially for ABs and TTs.

### 2.5. Chromatographic Fingerprints and Quantification of Terpenoid Components Entrapped in Lipo- and NLC-Nanoformulations by HPLC-DAD

**Figure 8:**
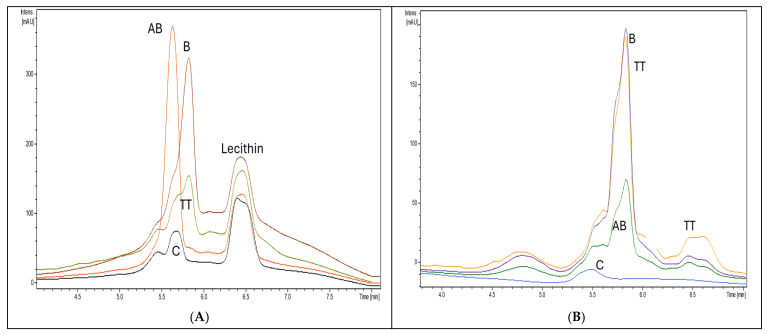
(**A**) Comparative, superposed HPLC-DAD chromatograms of Lipo-nanoformulations after entrapment of AB, B, and TT in Liposomes, compared to empty Lipo (C). (**B**) Superposed HPLC-DAD chromatograms of NLC-formulations after entrapment of AB, B, and TT in NLC, compared to empty NLC (C).

Figure 8 includes the superposed HPLC-DAD chromatograms (recorded at 212 nm) of the Lipo-nanoformulations by the solubilization of the retentate after the entrapment of AB, B, and TT in Liposomes, compared to empty Liposomes (Figure 8A) and solubilized NLC retentates after the entrapment of AB, B, and TT in NLC, compared to empty NLC (Figure 8B).

The HPLC-DAD fingerprints of the Lipo-formulations show the presence of lecithin (Rt = 6.3–6.5 min), the main component of Liposomes, a shift of Rt values, with Lipo-AB having AB-Rt = 5.5 min, B-Rt = 5.8, and TT presenting a main peak similar to B (Rt = 5.8 min), a second one, as a shoulder, attributed to AB (Rt = 5.5 min), and a minor peak at Rt = 6 min attributed to L. The fingerprints of NLC-formulations were different: the Rt values for B and TT were similar and superposed, at Rt = 5.8 min; for AB, at 5.7–5.9 min; and TT had a more pronounced shoulder at 6–6.2 min. The large peak between 6.4–6.7 min corresponded to other lipid constituents of NLC after entrapment (presumably oxidized lipid derivatives), while the control NLC had only a small peak at 5.5 min.

**Figure 9:**
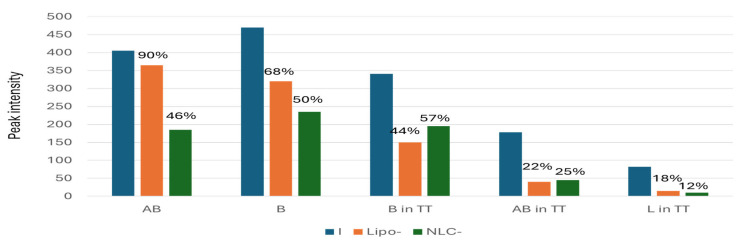
Comparative values of peak intensity (212 nm) for the initial concentrations (I) of pure standards of AB and B, as well as in TT, according to HPLC-DAD data and the peak intensity values recorded for the same terpenoids in the solubilized retentate after entrapment in Lipo- and NLC-formulations. The mean EE% values are marked on the top of columns.

According to the calibrations made with the B standard (see Supplementary File S1), the EE% values were determined by the solubilized retentate, in each case. Figure 9 shows comparatively the peak intensity values and the mean EE% values determined in each case.

The EE% values in Lipo- were superior to NLC-formulations, and ranged from 90% for AB to 68% for B. For TT, the major component B showed the highest EE% compared to AB and L, without significant differences of entrapment in Lipo- and NLC-formulations. These entrapment values were compared with the ones obtained by UV spectrometry and ATR-FTIR, as presented below (Section 2.6). Significant lower EE% values (*p* < 0.01) were noticed only for pure AB and B standards (46% and 50%, respectively).

### 2.6. Integration of Data Related to Entrapment Efficiency of the Three Methods

**Figure 10:**
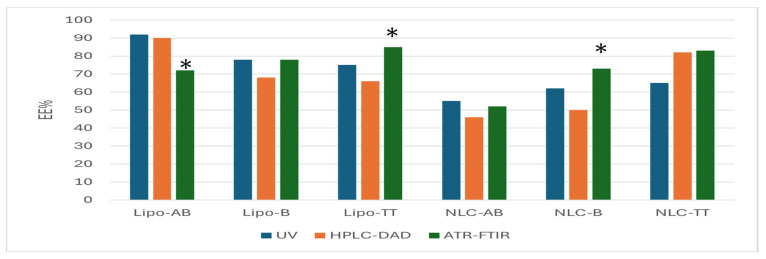
Comparative evaluations of EE%, as mean values, calculated according to the entrapment of AB, B, and TT in Lipo- and NLC-formulations. The mean values of EE% were obtained independently by the three methods: UV spectrometry, ATR-FTIR, and HPLC-DAD. * *p* < 0.01.

Figure 10 integrates the data obtained by UV spectrometry, HPLC-DAD, and ATR-FTIR regarding the entrapment of AB, B, and TT in Lipo- and NLC-formulations. The significant differences with *p* < 0.01 were labelled.

With some exceptions (Lipo-Ab, Lipo-TT, and NLC-B), the ATR-FTIR semi-quantitative evaluation of concentrations and EE% for the three terpenoids proved to have non-significant differences as compared to the accurate HPLC-DAD evaluation and also preliminary UV evaluation.

## 3. Discussion

In the current study, an extract rich in pentacyclic terpenoids (TTs) including betulin, betulinic acid, and lupeol from the outer bark of silver birch was obtained by successive extractions in organic solvents, modifying a procedure used before [33,34], and considering the improved solubility of betulin as a main component, in iso-propanol, ethyl acetate, and ethanol: DMSO [35]. The final concentrated TT extract reached the concentration of 28.35 mg total terpenoids /mL, according to a preliminary UV spectrometry. The entrapment efficiency was higher in Lipo- compared to NLC-formulations and superior for betulinic acid compared to betulin.

This rough evaluation was compared with the accurate quantification by HPLC-DAD, resulting in a total concentration of terpenoids expressed as 25.4 mg betulin equivalents/mL and a weight ratio of betulin:betulinic acid:lupeol of 55:35:10. By UHPLC-QTOF-ESI^+^-MS, betulin was identified by its main precursor with *m*/*z* = 443.3238 Da and a dominant fragment of 425.3495 Da (100%), while betulinic acid with a precursor ion of 457.3066 Da (100%) and a fragment of 439.3279 Da. Lupeol was identified by its specific fragments at 427.2653 Da, 409.2081 Da (100%), and 381.27 Da.

The standardized TT extract was used to build PEGylated liposomes and nano lipid carriers (NLCs) and compared with similar formulations containing pure AB and B (as positive controls). The procedures used for PEGylated liposomes were adapted from the classical hydration of lipid layers [36] to the ethanol injection method [30,37], while the composition of NLC was adapted from the other references [18,19]. Such formulations showed size ranges from 102 to 473 nm, with the NLCs having significantly higher diameters. In agreement with other references, a succession of analytical methods was applied to identify and quantify the components of the extract, either in organic solvents or in nanoformulations [38].

The use of ATR-FTIR as a semi-quantitative method for the evaluation of plant-based bioactive molecules, food and food supplements, or derived formulations for biomedical applications is well-documented [38]. Here, the ATR-FTIR spectroscopy of the TT extract was applied comparatively to pure betulin and betulinic acid, either as free molecules in a solvent mix or in nanoformulations. We identified their specific molecular fingerprints based on the main bands in two regions (850–1750 cm^−1^ and 2800–3400 cm^−1^). We identified 11 specific bands, with spectral features at 1716–1735 cm^−1^ attributable to the ν(C=O) mode, stretching vibrations of C=O identified from 1028 to 1180 cm^−1^, and characteristic alkyl stretching modes at 2823–2866 cm^−1^ and 2922–2945 cm^−1^. For a semi-quantitative evaluation, the calibration curves with pure betulin based on its ATR-FTIR spectra were obtained, and, according to the equations with the highest R^2^ (coefficient of determination), the wavelength at 2939.5 cm^−1^ was considered the most significant for the quantity. Accordingly, the encapsulation efficiency of all three terpenoids in nanoformulations was calculated and showed similar values (75 to 73%) of betulin entrapped in both the Lipo- and NLC-formulations, respectively. Meanwhile, the entrapment of betulinic acid and TTs in NLC-formulations had lower values, compared to liposomal formulations.

By the HPLC-DAD, the fingerprints and quantitation of betulin, betulinic acid, and lupeol in solvent and in Lipo- and NLC-formulations as well as their entrapment efficiency were determined. This method was able to identify each component, including excipients (lecithin in liposomes or lipids in NLC), and quantify the concentration of each terpenoid in nanoformulations. The entrapment values in Lipo- were superior to NLC-formulations, and ranged from 90% for AB to 68% for B. In the TTs, the major component B showed the highest entrapment comparative to AB and L, either in Lipo- and NLC-formulations. These values were compared with the ones obtained by UV spectrometry and ATR-FTIR, showing that ATR-FTIR offers a semi-quantitative evaluation which can be reliable compared to the accurate evaluation by HPLC. Therefore, ATR-FTIR spectroscopy is recommended to be used as a faster and cheaper method for the screening and semi-quantitative evaluation of free and entrapped terpenoids in nanoformulations, easier to be used directly for screening by ATR-FTIR. This method also offers the possibility of evaluating in a shorter time different recipes of nanoformulations as well as their stability and bioavailability.

## 4. Materials and Methods

### 4.1. Chemicals and Reagents

Betulin and betulinic acid (99% purity) were purchased from Roth GmbH Germany (Braunschweig, Germany). HPLC-grade solvents methanol, ethanol, iso-propanol, acetonitrile, formic acid, stearic acid, oleic acid, and triethanolamine were purchased from Merck (Darmstadt, Germany). PEG 2000, Triton X-100, Tween80, Lecithin (>98%), cholesterol were purchased from Sigma Aldrich (Merck Millipore, Darmstadt, Germany). Compritol 888ATO was provided from Gattefosse, (Saint-Priest, France).

### 4.2. Preparation of Birch Bark Extract TTs

The outer bark of silver birch (*Betula pendula*) was collected from forestry departments in Transylvania region in September 2023. The detached outer layer was dried for 24 h at 65 °C and stored in a dark desiccator. After grinding, 5 g of powder was washed 2× with petroleum ether to eliminate resins and then extracted successively with 2 × 100 mL mixture of iso-propanol:ethyl acetate, 1:1 (*v*/*v*) during 48 h at 50 °C and after evaporation, re-extracted in acetonitrile. Finally, this extract was evaporated under vacuum and re-extracted in a solvent mix of ethanol: dimethyl sulfoxide (DMSO), 3:1. This stock extract (TTs) was used in further experiments.

### 4.3. Preliminary Evaluation of TTs by UV–Vis Spectrometry

For a semi-quantitative evaluation of the total terpenoid content in TTs, 3 successive dilutions were made in the solvent mix, and the UV absorption was recorded, in the range 200–320 nm, using a spectrometer Perkin Elmer Lambda 25 (Waltham, MA, USA). In parallel, two calibration curves were built using pure betulinic acid (AB) and betulin (B), respectively, dissolved in the solvent mix of ethanol:dimethyl sulfoxide (DMSO) 3:1, in a range of 2–20 mg/mL. The calibration curves and equations were used to determine the concentration of terpenoids in TT extract, considering the triplicate measurement of UV absorption at 212 nm.

### 4.4. Preparation of PEGylated Liposomes Using the Ethanol Injection Method

Details about the procedure applied by ethanol injection method [30] were reported in our previous publications [31]. Shortly, a lipid phase included 16 mL soybean lecithin 6.25% in pure ethanol with an addition of 25 mg cholesterol. The aqueous phase included 20 mL phosphate buffer 0.01M pH 6.5, 40 µL Tween-80, and 20 mg PEG2000. Under magnetic stirring at 65 °C, the lipid phase was added dropwise to the aqueous phase, for min. 20 min. To remove residual ethanol, the emulsion was stirred gently for one hour. The liposomal suspension was ultrasonicated at highest amplitude, on a UP50H Compact Lab Homogenizer (Hielscher, Teltow, Germany). Empty control liposomes and liposomes containing the TTs (Lipo-TTs), pure AB (Lipo-AB), and B (Lipo-B) were prepared using the same procedure, as follows. The Lipo-AB and Lipo-B and were produced following this procedure: 50 mg AB and 50 mg B, respectively, were dissolved in 2 mL solvent mix and added to the lipid phase, just before mixing with the aqueous phase. A volume of 2 mL TT was added, similarly to the lipid phase.

### 4.5. Preparation of NLC-Formulations by Melt-Emulsification

To obtain the NLC-formulations, Compritol 888ATO, a standardized glycerol dibehenate mixed with stearic acid, oleic acid, Tween 80, and Triethanolamine, in a ratio 10:5:2.5:2.5 (weight ratios), was melted at 78 °C as described previously using the melt-emulsification procedure [31]. A dropwise addition of 20 mL hot ultrapure water phase (pH 7.2) to 2 g melted lipid mix (at 80 °C) was applied under ULTRA-TURRAX mixing at 20000 rpm, for 15 min. The hot emulsion was ultrasonicated for 10 min at highest amplitude using the UP50H Compact Lab Homogenizer (Hielscher). The resulting suspension was kept on ice for 15 min. To obtain NLC-TTs, NLC-AB, and NLC-B formulations, 50 mg AB, 50 mg B, and 2 mL TTs were dissolved in ethanol:DMSO (3:1), introduced in 20 mL hot water, and added dropwise to the melted lipid mix. The final suspension had a volume of 20 mL.

### 4.6. Entrapment Efficiency and Size Determination of PEGylated Liposomes and NLCs

To evaluate the entrapment efficiency (EE%) percentage of AB, B, and TTs in each formulation (Lipo- and NLC-), 10 mL from the Lipo- or NLC-suspensions were filtered under centrifugation (Hettich Rotofix 46 Centrifuge (Tuttlingen, Germany) at 4600 rpm, 30 min at 25 °C) through Amicon^®^ Ultra 15 mL Centrifugal Filter devices (Merck Millipore, Darmstadt, Germany) with 100 K cut-off. The retentate was collected after two successive washings. The retentate of Lipo- and NLC-suspensions after the filtration was resuspended up to the initial volume of 10 mL by addition of buffer and water, respectively. The sizes of nanoformulations were checked by DLS after 2 min vortex, and confirmed that no aggregation happened. To measure EE%, the retentate was dissolved in a solution of 0.1% Triton X-100 in ethanol and the absorptions (recorded at 212 nm by UV–Vis spectrometry) were compared to the ones of the initial suspensions dissolved in the same solvents, with absorption values of AB, B, and TTs. The entrapment efficiency was calculated by the formula EE% = (Aret/At) × 100, where At is absorption of initial suspension and Aret is absorption of retentate suspension. The sizes of all nanoformulations, before and after filtration, and the polydispersibility index (PDI) were also determined by laser diffraction technique using the Shimadzu SALD 2300 DLS instrument (Shimadzu, Japan) with the software Wing SALDII version 3.4.10, using a procedure, previously described [31].

### 4.7. Identification of TT Components by UHPLC-QTOF-ESI^+^-MS

This analysis was performed on a Bruker Daltonics MaXis Impact (Bruker GmbH, Bremen, Germany) device including a Thermo Scientific HPLC UltiMate 3000 system with a Dionex Ultimate quaternary pump delivery and ESI+-QTOF-MS detection, on C18 reverse-phase column (Kinetex, UPLC C18) (5 µm, 4.6 × 150 mm) at 25 °C, a flow rate of 0.8 mL/min, and an injection volume of 25 µL. The mobile phase was represented by a gradient of an eluent A (pure water containing 0.1% formic acid) and eluent B (Methanol:Acetonitrile:Isopropanol, 1:1:1, containing 0.1% formic acid). The gradient system consisted of 70% A (min 0), 30% A (min 4), 0% A (min 7), 30% A (min 10), and 70% A (min 13), followed by 2 min isocratic elution with 70% A. The total running time was 15 min. The MS parameters were set for a mass range between 100–1000 Da. The nebulizing gas pressure was set at 2.8 bar, the drying gas flow at 12 L/min, and the drying gas temperature at 300 °C. During each chromatographic run, a calibration with sodium formate was carried out. The control of the instrument and data processing used the specific software provided by Bruker Daltonics, namely, Chromeleon, TofControl 3.2, Hystar 3.2, and Data Analysis 4.2. The identification of each component was carried out by Data Analysys, considering the MS spectral analysis for each component and specific fragmentation. The Extract Ion Chromatogram was also used to confirm the specific fragments for B, AB, and L in the TT extract.

### 4.8. Fourier-Transform Infrared ATR-FTIR Spectroscopy

The ATR-FTIR spectra were recorded from 3700 to 800 cm^−1^ (4 cm^−1^ resolution and 64 scans) using a Spectrum 400 spectrometer (Shimadzu, Japan). The specific fingerprints and the absorbances corresponding to each wavenumber were evaluated and processed by the OPUS 5.5 software. The samples were analyzed in triplicate as standard solutions AB, B, and TTs in the solvent-mix solutions, as liquid suspensions (Lipo- and NLC-complexes) and after evaporation.

### 4.9. Quantitative Evaluation by HPLC-DAD

Pure standards of betulin and betulinic acid were used to build a calibration curve (1–10 mg/mL), to make quantitative evaluations (details in Supplementary File S1). We used aliquots of 0.5 g in triplicate, which were mixed with a mix of ethanol:DMSO 3:1 and homogenized under sonication 15 min. After 30 min, the extract was filtered by Millipore Nylon membrane (0.25 μm) (Burlington, MA, USA) and injected (10 μL) in the HPLC column (Acclaim 120, 100 mm × 2.1 mm × 5 μm (Glen Cove, NY, USA)) of an Agilent 1200 HPLC device (Santa Clara, CA, USA), with UV detection, applying a gradient of two mobile phases consisting of acetonitrile with 0.1% formic acid (A) and water with 0.1% formic acid (B). The gradient program was as follows: B = 55 to 15% (0–4 min), B = 30 (min 6), and B = 55% (min 8), with flow 0.3 mL/min at 25 °C. The detection was set at 210 and 280 nm. The concentrations of each component (AB and B) were calculated according to the calibration curve and expressed in mg per mL.

## 5. Conclusions

According to the general aims of medicinal chemistry, this study focused on the development of new bioactive molecules from natural resources, intending to find best formulations for their application in biomedicine. Based on previous studies, we developed rapid and convenient methods for the extraction of a subclass of pentacyclic triterpenoids, which offer good perspectives to be used as therapeutic drugs, either by parenteral, oral, or topical administration. Considering their limitations related to hydrophilicity and bioavailability, two types of nanoformulations were obtained and characterized. PEGylated liposomes and nanostructured lipid carriers were able to entrap betulin or betulinic acid pure standards and an organic extract from the outer birch bark, with different entrapment efficiencies. This objective was fulfilled, and we obtained nanoformulations in which the individual components were identified and quantified and compared with their organic solutions. The UV–Vis spectra were recorded as a preliminary investigation, with the advanced UHPLC-QTOF-ESI^+^-MS for the identification of components, the HPLC-DAD for the quantitative evaluation and calculation of the entrapment efficiency. The ATR-FTIR spectroscopy (which, despite its inability to provide full structural information, may be used as a fast, non-destructive technique to identify specific vibrational bands which can be correlated with the quantity of targeted molecules either in organic solvents or in nanosuspensions) was found to be a reliable method, giving results positively correlated with HPLC-DAD data. Therefore, the potential of ATR-FTIR to realize a fast “fingerprint” of samples was confirmed, with a semi-quantitative evaluation, being a non-destructive, cheaper, and easy-to-use technique. The results obtained can recommend the use of these nanoformulations, especially the liposomal ones, for further investigations in vitro and in vivo, which may confirm the efficacy of these terpenoids as therapeutic agents (antioxidant, antiviral, and anti-inflammatory) and especially as anticancer agents.

## Figures and Tables

**Table 1:** Specific vibrational bands identified in the TT extract compared to pure extracts of betulin (B) and betulinic acid (AB). Specific vibrations (*ν*-stretching; δ-bending; τ-torsion; and *ω*-wedging) were attributed to molecular vibrations found in terpenoids. The data were compared with published references.

Region (cm^−1^)	Wavenumbers for TTs/B/AB	Molecular Vibrations	References [15,31]			
			Raw Extract *Betula Pendula*	B	AB	L
2800–3400 cm^−1^	3363/3363/3360 w	*ν*(OH)	3362 s	3362 s	-	3309 s
	2939.5/2922/2945 vs	*ν*_as_(CH_3_) + *ν*_as_(CH_2_)	2940 vs	2968 vs	2943 vs	2982 s
	2866.2/2823/2852 s	+*ν*(CH_3_) + *ν*(CH_2_)	2867 s	2866 vs	2878 vs	2874 vs
860–1800 cm^−1^	1732.1 m; 1716.6 m	*ν*(C=O)	1709–1684, broad	1735 w, 1708 sh	1716 sh, 1681 vs	-
	1683.9 s; 1645.3 m	δ(CH_2_) + *ν*(C=C)	1642	1642 m	1642 m	1642 m
	1489.0 m; 1456.3 s	δ(CH_3_) + δ(CH_2_)	1484 m, 1452 s	1485 m, 1450 s	1451m	1453 m
	1373.3 m	δ(CH_3_) + δ(CH_2_)	1373 m	1373 m	1377 m	1374 m
	1184.3 m	*ν*(C-C) + δ(OH) +τ(CH_2_) + δ(CH)	1190 m	1190 m	1190 m	1190 m
	1028.1 s	ν(C-O) from CH_2_-OH, +δ(CH) + ρ (CH_3_, CH_2_)	1028 s	1032 ms	1043 m	1046 m
	881 m	*ω*(H-C-H) alkene	881 m	875 m	885 m	895 s

m—medium; s—strong; vs—very strong; as—asymmetric; w—weak.

**Table 2:** The equations of the calibration curves obtained for B standards (2, 4, 10, and 20 mg/mL) by ATR-FTIR spectroscopy at each of the 11 wavenumbers from ATR-FTIR spectra.

Wavenumber (cm^−1^)	Equation Curve	R^2^	Wavenumber (cm^−1^)	Equation Curve	R^2^
1028.1	0.0022x + 0.0084	0.960	1683.9	0.0032x + 0.0115	0.931
1184.3	0.001x + 0.0176	0.993	1716.6	0.0005x + 0.0342	0.627
1373.3	0.0017x + 0.0002	0.973	1732.1	0.0005x + 0.0023	0.452
1456.3	0.002x + 0.0144	0.883	2866.2	0.0021x + 0.0136	0.988
1489.0	0.0008x + 0.0134	0.768	2939.5	0.0034x + 0.0199	0.992
1645.3	0.0003x + 0.0209	0.396			

## Data Availability

The original contributions presented in this study are included in the article/supplementary material. Further inquiries can be directed to the corresponding authors.

## Supplementary Materials

The following supporting information can be downloaded at: https://www.mdpi.com/article/10.3390/ph18010033/s1, Supplementary File S1. HPLC-DAD chromatograms for pure standards (AB and B) at 2.5 mg/mL and TTs at two dilutions (2.5 and 1.25 mg/mL). Calibration curve of betulin (B) by HPLC-DAD recorded at 280 nm. Supplementary File S2. A. ATR-FTIR spectra (850–1750 cm^−1^ and 2800–3600 cm^−1^) of pure triterpenoid standards of AB and B at 2.5 mg/mL each, compared to TT extract. The spectra were recorded in EtOH:DMSO (3:1) solvent mix, before evaporation (s) and after evaporation. B. ATR-FTIR spectra of liposomal (LIPO) suspensions entrapped with AB, B, and TTs, before (s) and after evaporation. C. ATR-FTIR spectra of NLC-suspensions entrapped with AB, B, and TTs, before (s) and after evaporation. Supplementary File S3, Calibration curves based on FTIR-ATR spectra of Betulin (A-D) at four successive concentrations (2, 4, 10 and 20 mg/mL) in Ethanol: DMSO, 3:1. The curves were built for main peaks identified and attributed to characteristic vibrations of triterpenoid molecules: 1028.1, 1184.3, 1373.3, 1456.3,1489.3, 1645.3, 1683.33,1716.6, 1732.1, 2866.2, 2939.5 cm^−1^.
