# Supplementary material for: Synthesis and Characterization of PEGylated Liposomes and Nanostructured Lipid Carriers with Entrapped Bioactive Triterpenoids: Comparative Fingerprints and Quantification by UHPLC-QTOF-ESI+-MS, ATR-FTIR Spectroscopy, and HPLC-DAD
Source: Pharmaceuticals (Basel). 2024 Dec 31;18(1):33. doi: 10.3390/ph18010033 (PMC11768173; doi:10.3390/ph18010033)
Supplement: Supplementary file 1 [file pharmaceuticals-18-00033-s001.zip › Suppl. file S2 FTIR comp free, Lipo si NLC in sol vs evap.pdf]

Suppl file S2. **A.** ATR-FTIR spectra (850-1750 cm^{-1} and 2800-3600 cm^{-1}) of pure triterpenoid standards AB and B at 2.5 mg/ml each, comparative to TT extract. The spectra were recorded in EtOH:DMSO (3:1) solvent mix, before (s) and after evaporation. **B.** ATR-FTIR spectra of liposomal (Lipo-) suspensions entrapped with AB, B and TT, before (s) and after evaporation. **C.** ATR-FTIR spectra of NLC suspensions entrapped with AB, B and TT, before (s) and after evaporation.

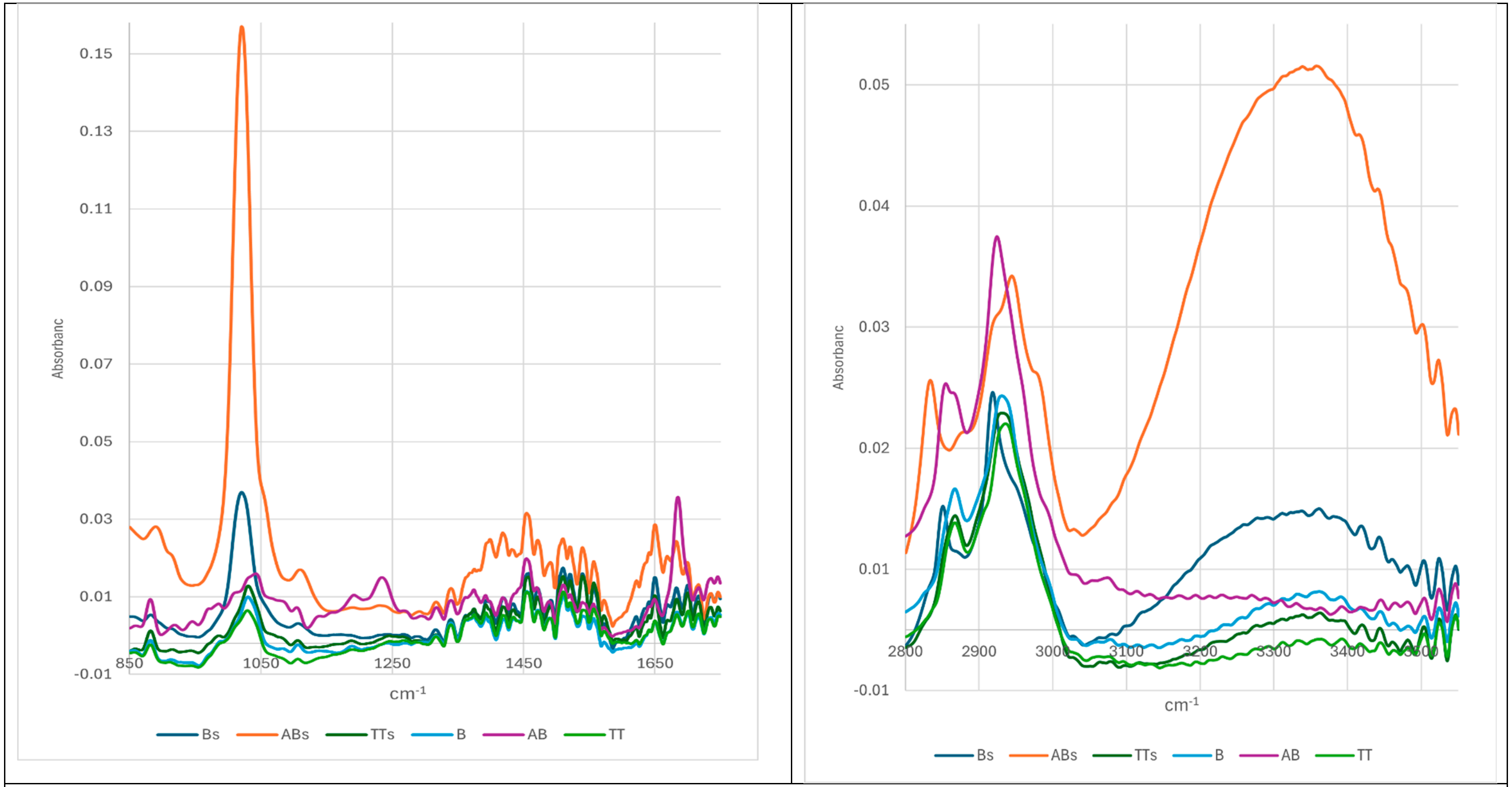

S2A.

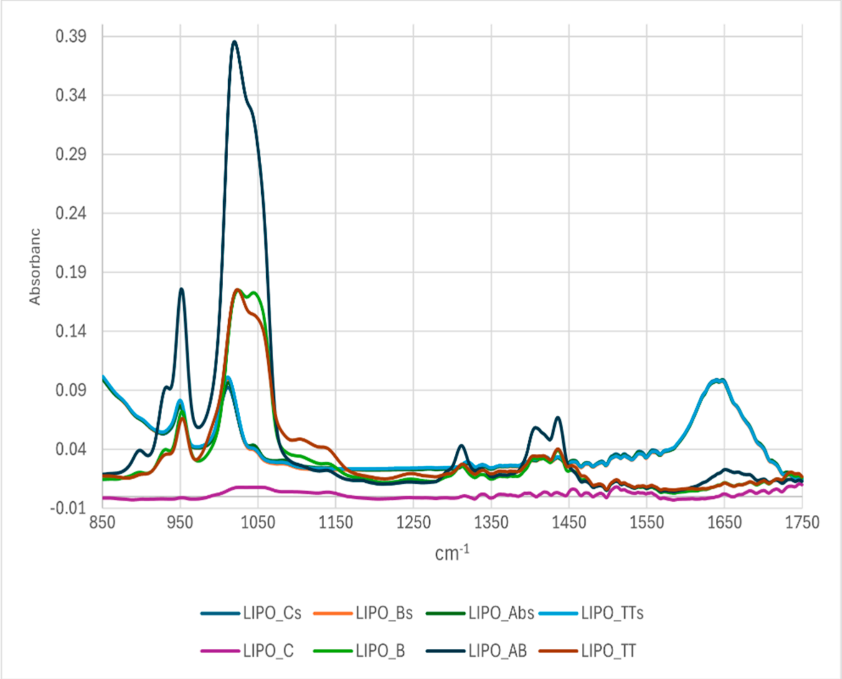

0.39
0.34
0.29
0.24
0.19
0.14
0.09
0.04
-0.01
Absorbanc
850
950
1050
1150
1250
1350
1450
1550
1650
1750
cm-1
LIPO_Cs
LIPO_Bs
LIPO_Abs
LIPO_TTs
LIPO_C
LIPO_B
LIPO_AB
LIPO_TT

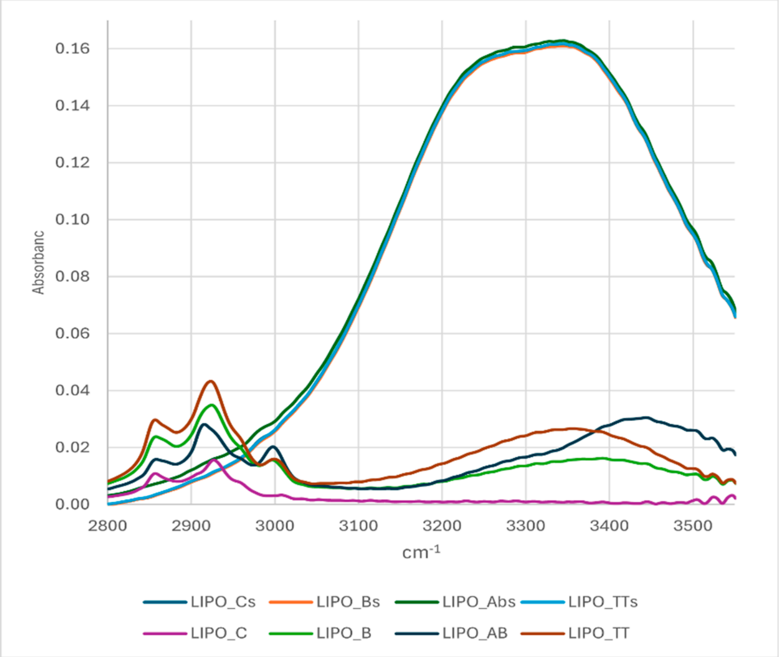

0.16
0.14
0.12
0.10
0.08
0.06
0.04
0.02
0.00
Absorbanc
2800
2900
3000
3100
3200
3300
3400
3500
cm-1
LIPO_Cs
LIPO_Bs
LIPO_Abs
LIPO_TTs
LIPO_C
LIPO_B
LIPO_AB
LIPO_TT

S2B.

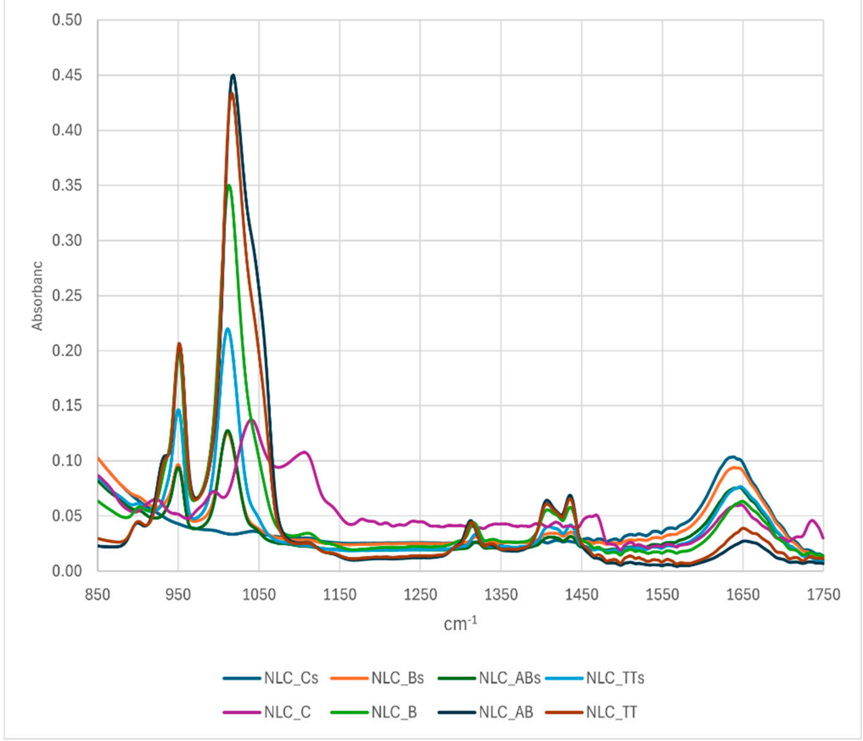

Absorbanc
cm-1
NLC_Cs
NLC_Bs
NLC_ABs
NLC_TTs
NLC_C
NLC_B
NLC_AB
NLC_TT

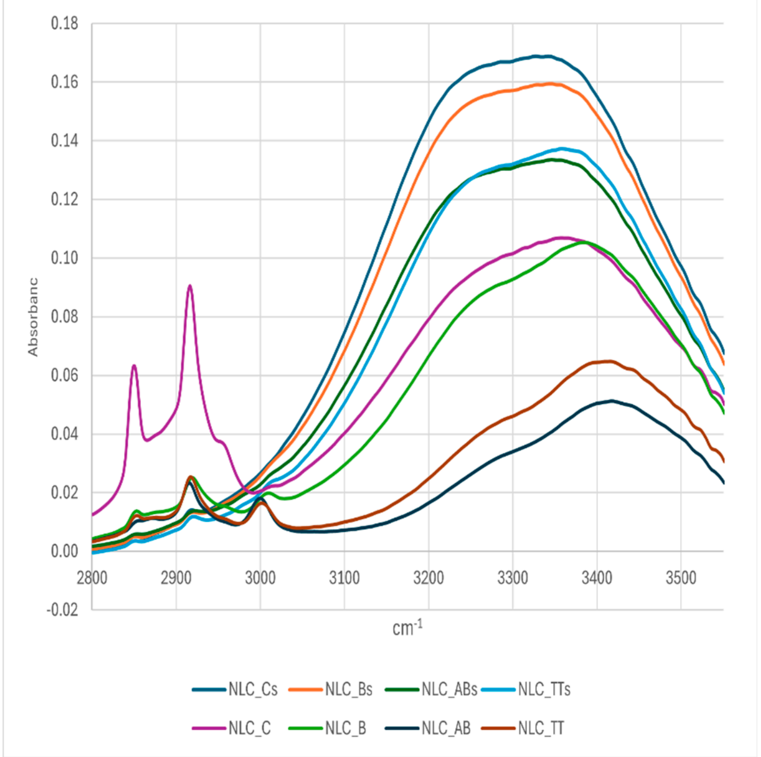

Absorbanc
cm-1
NLC_Cs
NLC_Bs
NLC_ABs
NLC_TTs
NLC_C
NLC_B
NLC_AB
NLC_TT

S2C.
