# Supplementary material for: Synthesis and Characterization of PEGylated Liposomes and Nanostructured Lipid Carriers with Entrapped Bioactive Triterpenoids: Comparative Fingerprints and Quantification by UHPLC-QTOF-ESI+-MS, ATR-FTIR Spectroscopy, and HPLC-DAD
Source: Pharmaceuticals (Basel). 2024 Dec 31;18(1):33. doi: 10.3390/ph18010033 (PMC11768173; doi:10.3390/ph18010033)
Supplement: Supplementary file 1 [file pharmaceuticals-18-00033-s001.zip › Suppl. file S3 Calibration curves FTIR for free molecules AB and B.pdf]

Supplementary file S3. Calibration curves based on FTIR-ATR spectra of Betulin (A-D) at four successive concentrations (2, 4, 10 and 20 mg/ml) in Ethanol: DMSO, 3:1. The curves were built for main peaks identified and attributed to characteristic vibrations of triterpenoid molecules: 1028.1, 1184.3, 1373.3, 1456.3,1489.3, 1645.3, 1683.33,1716.6, 1732.1, 2866.2, 2939.5 cm^{-1}.

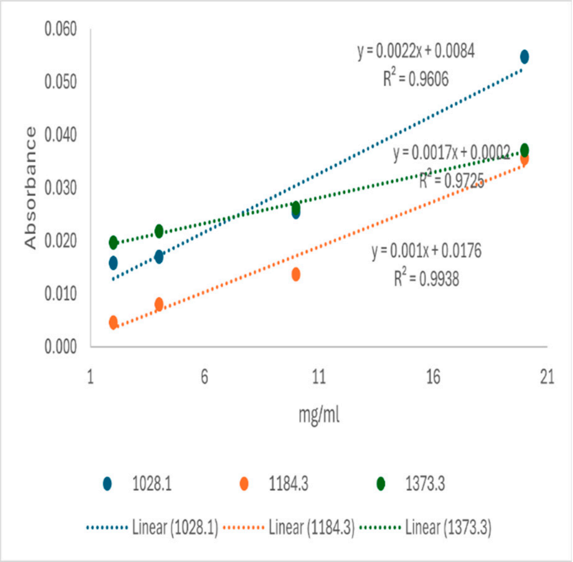

A.

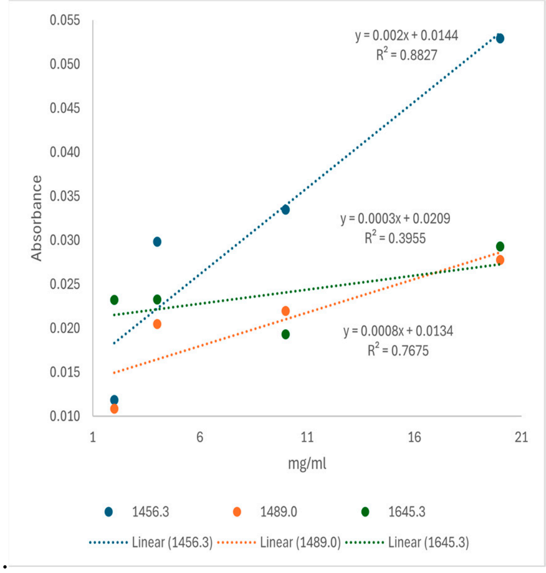

B.

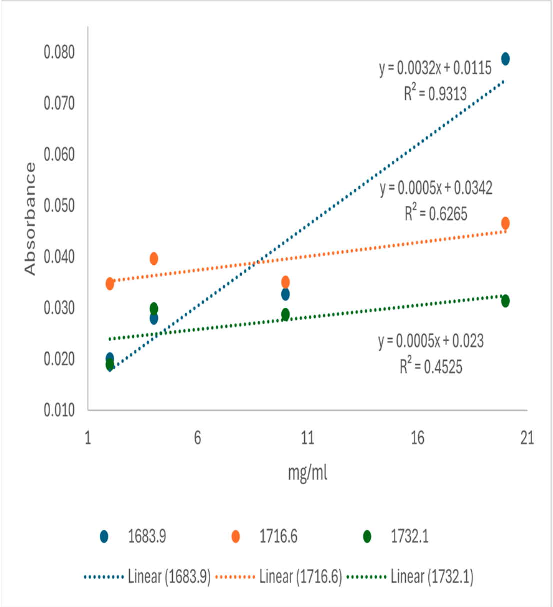

C.

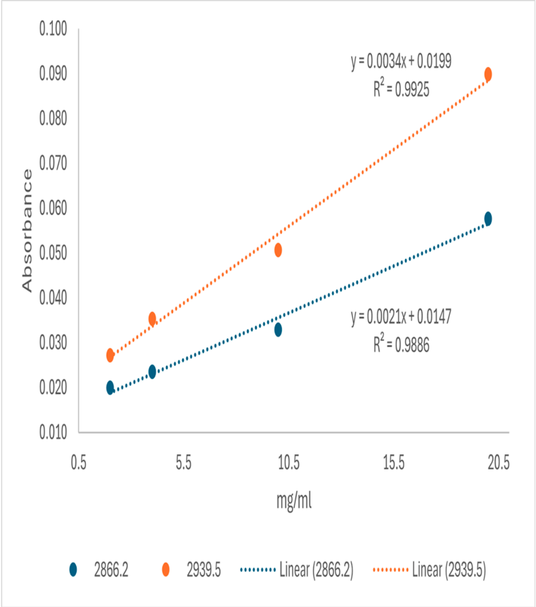

D.
