# Supplementary material for: Synthesis and Characterization of PEGylated Liposomes and Nanostructured Lipid Carriers with Entrapped Bioactive Triterpenoids: Comparative Fingerprints and Quantification by UHPLC-QTOF-ESI+-MS, ATR-FTIR Spectroscopy, and HPLC-DAD
Source: Pharmaceuticals (Basel). 2024 Dec 31;18(1):33. doi: 10.3390/ph18010033 (PMC11768173; doi:10.3390/ph18010033)
Supplement: Supplementary file 1 [file pharmaceuticals-18-00033-s001.zip › Suppl.File S1 Calibration curve HPLC-DAD.pdf]

Supplementary File S1. HPLC-DAD chromatograms for pure Standards (AB and B) at 2.5 mg/ml and TTs at two dilutions (2.5 and 1.25 mg/ml). Calibration curve of betulin (B) by HPLC-DAD recorded at 212 nm.

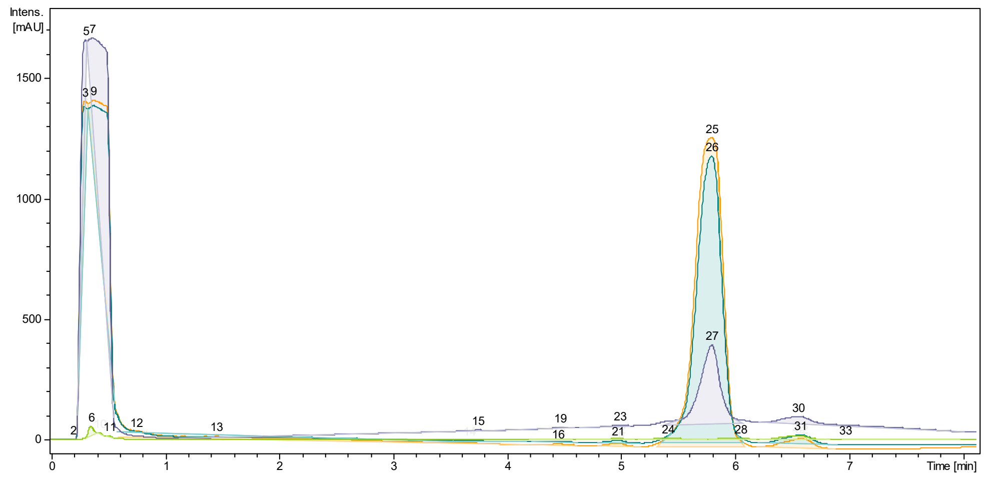

A.

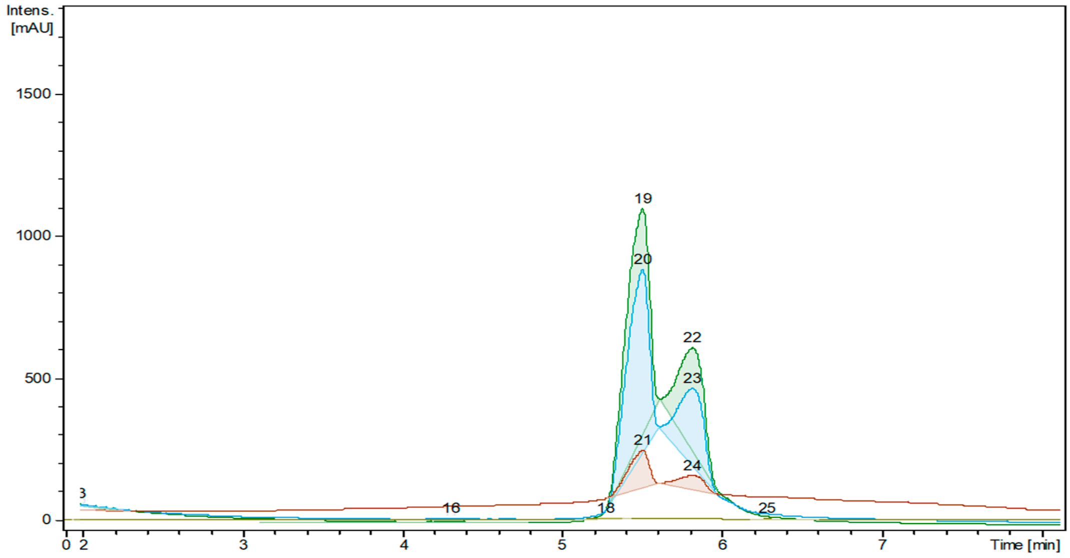

B

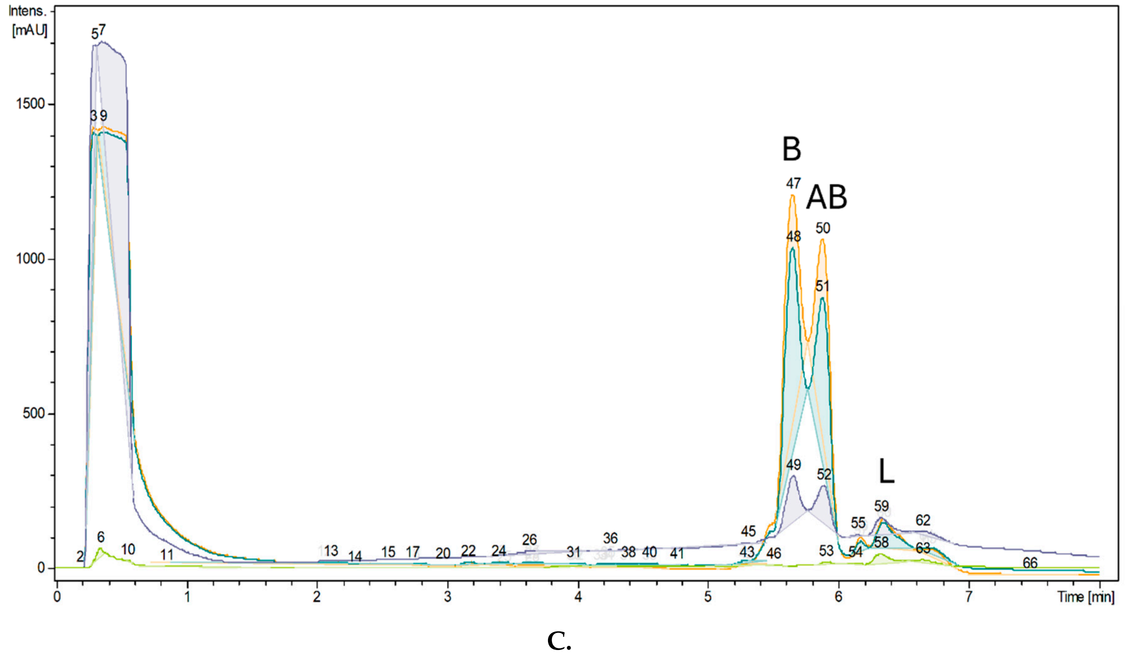

Figure S1. Comparative HPLC-DAD chromatograms recorded at 212 nm for AB (**A**) and B + AB (**B**) pure standards at three successive concentrations and a chromatogram of TTs (**C**) . B-Rt = 5.5, AB-Rt= 5.9 min and Rt= 6.35 min. The peak area ratio of B: AB: L was determined to be 55: 35: 10 according to peak areas ratios.

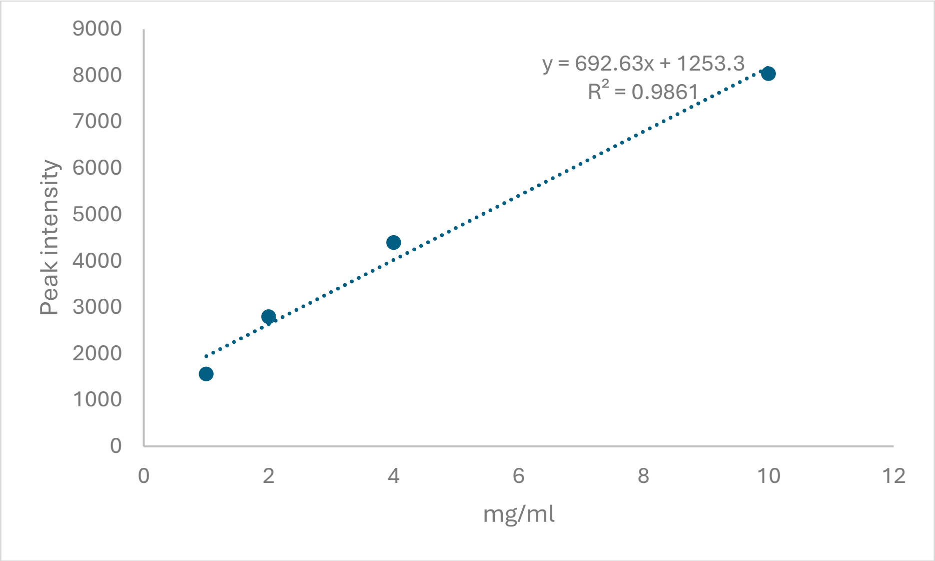

Calibration curve for a solution of pure B in EtOH:DMSO (3:1) (1 to 10 mg/ml) according to HLC-DAD analysis withdetection at 212 nm.
